# Neuronal control of peripheral insulin sensitivity and glucose metabolism

**DOI:** 10.1038/ncomms15259

**Published:** 2017-05-04

**Authors:** Johan Ruud, Sophie M. Steculorum, Jens C. Brüning

**Affiliations:** 1 Department of Neuronal Control of Metabolism, Max Planck Institute for Metabolism Research, Gleueler Strasse 50, 50931 Cologne, Germany; 2 Policlinic for Endocrinology, Diabetes and Preventive Medicine (PEDP), University Hospital Cologne, Kerpener Strasse 26, 50924 Cologne, Germany; 3 Excellence Cluster on Cellular Stress Responses in Aging Associated Diseases (CECAD) and Center for Molecular Medicine Cologne (CMMC), University of Cologne, Joseph-Stelzmann-Strasse 26, 50931 Cologne, Germany; 4 National Center for Diabetes Research (DZD), Ingolstädter Land Strasse 1, 85764 Neuherberg, Germany

## Abstract

The central nervous system (CNS) has an important role in the regulation of peripheral insulin sensitivity and glucose homeostasis. Research in this dynamically developing field has progressed rapidly due to techniques allowing targeted transgenesis and neurocircuitry mapping, which have defined the primary responsive neurons, associated molecular mechanisms and downstream neurocircuitries and processes involved. Here we review the brain regions, neurons and molecular mechanisms by which the CNS controls peripheral glucose metabolism, particularly via regulation of liver, brown adipose tissue and pancreatic function, and highlight the potential implications of these regulatory pathways in type 2 diabetes and obesity.

## Abstract (web summary)

The brain controls peripheral glucose metabolism, for example by modulating hepatic gluconeogenesis or by regulating glucose uptake into brown adipose tissue. Here, the authors review the brain regions, neurons and molecular mechanisms involved in these processes, and discuss their relevance to disease.

More than one third of the adult population is obese in many countries, including newly industrialized states, making obesity a global human health problem^1^. Obesity is often accompanied by insulin resistance (the condition when cells fail to respond to insulin) and glucose intolerance (the inability of cells to clear glucose from the blood stream after a glucose load), the prevalence of which are estimated to advance as the number of obese individuals continues to increase^2^. Obesity constitutes an important risk factor not only for the development of type 2 diabetes (T2D), but also for cardiovascular disease and even certain types of cancer, all of which ultimately reduce life expectancy^3^,^4^.

Insulin resistance and glucose intolerance result in a disturbed glucose homeostasis, a state that describes the inability to maintain stable glucose levels (euglycemia). Maintenance of euglycemia is controlled via the tightly balanced action of hormones, such as cortisol and glucagon, which increase blood glucose concentrations; insulin, on the other hand, is the only hormone identified that is capable of clearing glucose from the blood. Insulin acts on the insulin receptor (IR), a membrane bound tyrosine kinase^5^, which lowers blood glucose concentrations by promoting glucose uptake, while also suppressing hepatic glucose production (HGP) (Fig. 1).

Over the past 20 years, novel molecular mechanisms linking obesity and the development of insulin resistance have been deciphered, including obesity-associated inflammation, deregulated endoplasmic reticulum-stress regulation, mitochondrial dysfunction and lipotoxicity. Whereas the field has largely focused on direct effects of obesity-associated alterations in peripheral tissues such as liver, skeletal muscle and adipose tissue (Fig. 1), the role of the central nervous system (CNS) as a regulator of energy fluxes across different organs and with effects on glucose homeostasis has not received the same attention. Recently, an important role for the CNS in the regulation of peripheral insulin sensitivity and glucose homeostasis has been unravelled. We review the progress made in this research field and place particular emphasis on the central control of liver and brown adipose tissue (BAT) as well as pancreatic islet function in control of glucose metabolism. We provide an update on the key brain regions, neurons and molecular mechanisms in these neurons and the downstream neurocircuitries identified, as well as outline relevant peripheral mediators that act on the these brain circuits in the control of glucose homeostasis. We also review recent literature on how obesity perturbs CNS-dependent control of glucose metabolism, and highlight the potential clinical relevance of these regulatory CNS pathways in T2D.

## Key CNS sites in control of glucose metabolism

Solid evidence for a role of CNS circuits in regulating systemic glucose homeostasis dates back to the 1850s (Box 1). Today, a large literature substantiates energy-regulatory capabilities of a plethora of areas in the rodent brain (Fig. 2). Among those, several nuclei residing in the hypothalamus stand out, of which the arcuate nucleus (ARH), the ventromedial nucleus (VMH) and lateral hypothalamic area (LHA) have received most attention. We now recognize a neuroregulatory network governing control over feeding, peripheral insulin sensitivity and glucose metabolism extending beyond the ARH, VMH and LHA (Table 1). These regulatory centres also include a number of extra-hypothalamic nuclei, such as sensory and integrative clusters in the hindbrain^6^,^7^, as well as autonomic, parasympathetic and sympathetic preganglionic brainstem neurons^8^,^9^. Owing to the application of cell-specific chemogenetic and optogenetic techniques^10^,^11^, several of these nuclei were initially documented to orchestrate the behavioural and autonomic repertoire that controls feeding (Table 2) and some of these neurons have more recently been assigned gluco-regulatory properties beyond and even independent of their food intake-regulatory function.

### The arcuate nucleus of the hypothalamus

The ARH is located at the floor of the third ventricle, leveling with the base of the pituitary stalk (a funnel of nerves connecting the brain with the pituitary gland) and bridges with the median eminence. ARH neurons sense peripheral substances that signal the energy state of the organism. Accordingly, pro-opiomelanocortin (POMC), and agouti-related peptide (AgRP)/neuropeptide Y (NPY), expressed in two anatomically neighbouring and molecularly defined neuronal populations in the ARH (Fig. 2) are vital for the control of energy homeostasis in that they have profound effects on appetite and feeding behaviour (Box 2). In line with their ability to integrate peripheral signals and adapt their electrical activity according to energy availability, chronic manipulations of hormonal and nutrient signalling in POMC and AgRP neurons affect glucose metabolism in peripheral tissues^12^,^13^. However, whether POMC or AgRP neuron firing acutely controls glucose metabolism was not established until recently. Using cell-specific excitatory techniques, acute activation of AgRP neurons was found to impair systemic insulin sensitivity and glucose tolerance after acutely raising insulin or glucose in the bloodstream^14^. Specifically, AgRP-neuron activation halved insulin-stimulated glucose uptake selectively into BAT, likely through re-programming the gene expression profile towards a myogenic signature^14^. The most strongly upregulated gene in BAT was myostatin, a molecule previously linked to abnormal glucose metabolism^15^. Indeed, acute induction of myostatin partially explained the insulin resistance downstream of AgRP-neuron stimulation^14^.

Previous studies showed that acute activation of AgRP neurons reduces energy expenditure^16^, whereas mice genetically modified to lack AgRP neurons burn slightly more calories^17^, indicating a relationship between AgRP neurons and brown fat function. Consistent with these observations, acute activation of AgRP neurons decreased the activity in sympathetic nerves supplying BAT, and a lower β-adrenergic tone contributed to the development of systemic insulin resistance upon AgRP-neuron activation^14^. Pursuing the neurocircuitry downstream of AgRP-neuron driven insulin resistance using optogenetic circuit mapping, impaired insulin sensitivity and induced myostatin expression in BAT ensued when acutely stimulating fibres comprising AgRP^ARH→BNST^ projections, involving the ventrolateral subnucleus of the anterior BNST (AgRP^ARH→vlBNST^). Activating this projection in the vlBNST did not induce a feeding response however^14^. With respect to appetite control, activation of long- and short-range outputs from distinct subpopulations of AgRP neurons to several downstream sites is sufficient to evoke feeding alone. These observations point to a parallel and redundant organization of AgRP neuronal circuits that controls feeding behaviour^18^. As far as glucose homeostasis is concerned, one projection from AgRP neurons to the dorsomedial subnucleus of the aBNST controls feeding but not insulin sensitivity^14^, whereas one AgRP^ARH→vlBNST^ pathway controlling insulin sensitivity also encodes induction of BAT myostatin but not feeding. Although all AgRP neuron projections sites potentially controlling systemic glucose metabolism have not yet been probed, the data available thus suggest that peripheral insulin sensitivity is controlled by less redundant AgRP neuron circuits compared to those in control of feeding behaviour.

By contrast, acute activation of POMC neurons had no effect on glucose metabolism in these studies^14^, suggesting that acute AgRP neuron activation controls peripheral insulin sensitivity without interfering with the melanocortin pathway. Experiments defining melanocortin-dependent feeding behaviour have shown that the hypophagia from stimulating POMC neurons is prevented in *A*^*y*^ mice, in which AgRP constitutively blocks melanocortin signalling. By contrast, the hyperphagia from activating AgRP neurons is intact in *A*^*y*^ mice^19^, and melanocortin receptor blockade cannot prevent the hypophagic response upon AgRP neuron ablation^20^. Taken together, AgRP neurons may similarly control glucose metabolism independently of melanocortin signalling. To this end, AgRP neurons also synthesize NPY and GABA, and whereas AgRP through its action on MC4Rs is sufficient to trigger sustained but delayed increase in food intake, both NPY and GABAergic signalling contribute to the rapid hyperphagia observed upon AgRP neuron excitation^21^,^22^. AgRP neurons may thus govern control over glucose metabolism through NPY, GABA receptor signalling, or a combination of both. Interestingly, induced NPY expression specifically from the ARH reduces energy expenditure and decreases BAT thermogenesis via NPY1-receptor signalling in key nuclear relay stations, including the locus coeruleus, solitary tract nucleus and ventrolateral medulla in the hindbrain, some of which modulate sympathetic outflow to BAT^23^. These observations indicate that NPY-receptor signalling downstream of AgRP neurons may explain some of the effects on brown fat physiology exerted by AgRP neurons, and possibly systemic insulin sensitivity. Thus, further studies will have to address if NPY and/or GABA signalling exert the rapid glucose regulatory function of AgRP neurons.

Finally, although acute activation of POMC neurons was ineffective in affecting glucose metabolism in these studies, it is noteworthy that a recent study reported that chemogenetic activation of POMC^ARH^ neurons markedly and rapidly (within minutes) increases BAT temperature by several degrees^24^, demonstrating that POMC^ARH^ neurons promote BAT thermogenesis. The reasons why POMC-positive ARH cells potently affect BAT temperature without clear effects on insulin sensitivity are currently unknown, and future studies will be needed to address the nature of this divergence.

### The ventromedial nucleus of the hypothalamus

The brain launches an adaptive and protective counter-regulatory response when glucose levels fall out of range. The VMH (Fig. 2) is known to be particularly important for this counter-regulatory response^25^. Glucose-sensing mechanisms in VMH neurons, which are excited or inhibited by local changes in ambient glucose concentrations, and exert important roles in maintaining energy balance and glucose metabolism, have been carefully analysed, in particular in VMH neurons expressing steroidogenic factor-1 (SF-1)^26^ and/or the gene encoding glucokinase. In mice that are hypoglycemic owing to a high dose of insulin, the ability to normalize glycaemia fails when SF-1 neurons are optogenetically inhibited, as the anticipated rebound from hypoglycemia elicited by insulin is attenuated^27^. In turn, optogenetic activation of SF-1 neurons increases blood glucose, and causes profound hyperglycaemia when blood glucose levels are elevated either by stimulating HGP or by injecting glucose into mice^27^. The differential responses may stem from a failure of stimulating glucagon and corticosterone release (when SF-1 neurons are inhibited), or from the inability to balance glucagon and corticosterone secretion and control HGP (when SF-1 neurons are stimulated). It is conceivable that photostimulation of SF-1-expressing neurons mimics a state of glucodeprivation in the VMH since they stimulate the counter-regulatory response to hypoglycemia, including effects on pancreas and liver. Further expanding our understanding of the neural network for counterregulation, stimulating SF-1^VMH→aBNST^ axonal projections was recently shown to result in increased plasma levels of glucagon and corticosterone and hyperglycaemia^27^. Thus, a defined circuit spanning from glucose-sensing VMH neurons to the aBNST specifically regulates expression of key genes for hepatic gluconeogenesis and influences the abundance of counter-regulatory hormones striving to restore glycaemia.

In another study, investigators used radiowaves to manipulate glucokinase-expressing VMH neurons engineered to respond to an electromagnetic field, and showed that activation of VMH neurons robustly elevates blood glucose and glucagon concentrations in the circulation as well as drives the expression of key hepatic gluconeogenic genes, whereas inhibition quells these responses^28^. These findings further substantiate a role for the VMH in the control of peripheral glucose metabolism, and the authors describe a novel technique, dubbed magnetogenetics, to affect neuronal activity through a genetically encoded fusion protein between the iron-binding protein ferritin and a thermo-sensitive ion channel protein. Although the paper describes a way to remotely manipulate the electrical activity of neurons in mice with a very clear outcome^28^ and whereas a string of recent articles report the successful use of magnetogenetics, the way the underlying operative mechanism biophysically works is unclear and has turned into a subject of debate^29^.

To ensure that the field strength was adequate to affect neuronal activity, while permitting assessments of its impact on glucose metabolism *in vivo*, the mice had to be anesthetized in those studies^28^. Although the findings obtained from manipulating VMH neurons were the expected, whether exactly the same outcome is present in awake mice could not be proven with the confines of the method, as narcosis might have intrinsic effects on neural activity and glucose homeostasis. Thus, refinements of the necessary equipment for electromagnetics is required for large-scale use and to set the stage for further exciting discoveries. Moreover, future studies are encouraged to define the precise mechanism of magnetogenetics.

Although recent research has provided a wealth of information, the functional organization of the neurocircuity influencing counter-regulatory mechanisms of glycemic control remains to be better understood, and electromagnetics is hoped to provide more answers on the neuroendocrine components and architecture contributing. While the aBNST has surfaced as a key integrative glucoregulatory node, the details about this system remain to be specified. Specifically, which descending neural network downstream of the aBNST, tethering it do BAT glucose utilization, insulin sensitivity and counter-regulatory responses, as well as the exact cellular phenotype of the crucial aBNST neurons are issues that clearly call for additional study.

### The preoptic area and lateral hypothalamic area

Located along the midline of the anterior hypothalamus, the preoptic area (PoA) is situated closely below the anterior commissure (where nerve bundles pass between the two brain hemispheres) and above the optic chiasm (where optic nerve fibres from the retinas cross between the two hemispheres) (Fig. 2). The PoA regulates BAT heat production, a process that depends on the metabolism of significant amounts of glucose and triglycerides^30^,^31^,^32^. Nevertheless, the thermoregulatory function of this brain region has been primarily studied in the context of fever, which is driven by prostaglandin signalling in the median preoptic subnucleus^33^ and activates brown fat thermogenesis via a neural pathway including the rostral raphe pallidus (Fig. 2).

Surgical or electric manipulations of the LHA neurons over 50 years ago were shown to control food intake. We now know that a part of this effect is explained by an inhibitory synaptic innervation from the BNST to glutamatergic LHA neurons, eliciting voracious feeding in mice that are already satiated when optogenetically manipulated^34^. In food-deprived animals, inhibiting this input onto the LHA conversely suppresses feeding^34^. Furthermore, projections to the LHA from AgRP neurons impair systemic insulin sensitivity when activated^14^. Whether impairments in insulin sensitivity induced by the AgRP^ARH→LHA^ circuit also involve excitatory LHA neurons is currently unknown.

So far, recent observations point toward a critical role for MC4R signalling in the LHA in control of glucose homeostasis^35^. By reconstituting MC4R expression specifically in LHA neurons of obese mice carrying a null MC4R allele (MC4R^LHA^), Morgan *et al*. were able to improve glucose tolerance and glycaemia in both normal chow and high-fat diet (HFD)-fed mice independent of changes in body weight, adiposity or insulin concentrations^35^. Activation of the MC4R using an α-melanocyte stimulating hormone (α-MSH) analogue in mice with MC4Rs re-expressed in the LHA increased glucose uptake specifically into brown fat; this effect correlated with subtle increments in glucose transporter 4 (*GLUT-4*) gene expression and upregulation of a thermogenic gene expression programme in BAT^35^. Consistent with the idea that MC4R^LHA^ signalling facilitates BAT glucose utilization via the sympathetic nervous system, nerves innervating BAT showed normal spiking responses to a MC4R agonist in mice carrying a reactivated MC4R gene in the LHA, in contrast to the nerves in obese whole-body MC4R knockouts that were insensitive, and surgically eliminating BAT from sympathetic input furthermore impaired the improved glucose tolerance obtained from MC4R^LHA^ reactivation^35^. Thus, MC4R^LHA^ signalling activates sympathetic outflow to BAT, and intact sympathetic control over BAT glucose uptake is required to rescue the glucose tolerance when the MC4R is gone in every cell but in LHA neurons, as judged from this comprehensive study in mice^35^.

## Brown fat activity and humans

In humans, the quantity of BAT correlates inversely with BMI, BAT is highly responsive to cold and diet exposure, an adaptive response that is reduced in obese and overweight subjects, and insulin^36^,^37^,^38^,^39^,^40^. There is evidence that BAT is less active in diabetics^41^ and that BAT activation improves whole-body glucose homeostasis and insulin sensitivity^42^. Such observations have fostered the notion that strong actuators of BAT activity could be used to treat obesity and diabetes.

Brown fat function is often studied under cold conditions, a state that does not allow capturing whether BAT plays a role in glucose metabolism at euthermia. To measure whether metabolic activity in human BAT affects blood glucose levels over time and depending on feeding state and circadian rhythm, Lee and colleagues measured the temperature profile of the skin overlying supraclavicular BAT as a surrogate of conventional fluorodeoxyglucose positron emission tomography (FDG-PET) imaging^43^. At thermoneutrality, supraclavicular BAT temperature progressively rose during a glucose load, indicating that BAT utilizes glucose. The authors also observed a noteworthy rhythmicity in glucose uptake into human brown adipocytes, especially after insulin stimulation, together with oscillating trafficking of GLUT-4 to the plasma membrane, which mirrored the fluctuations in glucose uptake and generated heat^43^. In humans (normal weight, non-diabetic men in their mid-twenties) with larger than average active BAT depots, changes in BAT thermogenesis predicted subcutaneous blood glucose levels, whereas BAT thermogenic activity responded to systemic changes in glycaemia in individuals with comparatively small amounts of BAT^43^. Notably, men devoid of supraclavicular BAT exhibited the largest glycemic variability. Conceivably, human BAT glucose utilization is linked to thermogenesis, and BAT shows a glucose-responsive rhythm entrained by circadian oscillations in GLUT-4 in a similar manner as mechanisms coordinating body temperature rhythmicity and responses to cold^44^. In light of these findings, whether greater fluctuations in glucose levels as a consequence of the amount of functionally active BAT pre-dispose for diabetes warrant further investigations.

## Hormonal signalling to the brain and effects on glucose metabolism

Afferent hormonal and nutritional cues provide feedback signals to the brain that are crucial for systemic glucose homeostasis. On the other hand, efferent signalling from the brain to peripheral tissues is promoted via the autonomic nervous system, for example to control HGP, BAT activity and pancreatic hormone secretion (Fig. 3). Although Claude Bernard's (Box 1) work was an exciting starting-point, the idea that the brain controls peripheral glucose metabolism was largely ignored by the scientific community for a long time. However, several discoveries made in the past 20 years have reignited interest in this concept. Firstly, activation of the IR, which is widely expressed throughout the CNS, was shown to curb eating. Secondly, manipulation of key IR signalling components (such as PI3 kinases), activation of neuronal ATP sensitive potassium channels^45^, or depletion of functional IRs from the brain^46^, affect not only energy homeostasis but also systemic glucose metabolism. In humans, insulin quenches HGP via the same class of potassium channels (K_ATP_) as it does in rodents^47^. Insulin activates K_ATP_ channels in a PI3 kinase-dependent manner resulting in hyperpolarization of neurons^13^,^48^. However, how various hypothalamic neurons respond electrically to insulin might differ, as exemplified by the recent findings that insulin can excite POMC neurons via activation of canonical transient receptor potential channels in a PI3 kinase-dependent manner^49^. Similarly, insulin promotes PI3 kinase signalling in melanin-concentrating hormone (MCH) neurons in the LHA and increases their excitability^50^. Physiologically, insulin-dependent activation of these neurons impairs locomotor activity and glucose homeostasis by controlling hepatic insulin sensitivity and HGP in mice fed a HFD. Given that the phenotypic alterations dependent on IR signalling in MCH neurons were observable in HFD-fed mice but not lean mice fed a normal mouse chow suggest that this mechanism is engaged only during conditions when insulin levels rise. Consistent with this, HFD feeding associated with hyperinsulinemia increases PI3-kinase activity in MCH neurons via the IR^50^.

The insulin-dependent effects on MCH-expressing cells supports the existence of selective hormone resistance, which describes the occurrence of insulin resistance in cell types within the CNS with simultaneous retained or even over-activated insulin action in other CNS cell types. Indeed, the manifestation of selective CNS resistance to insulin represents a rule rather than exception^26^. In fact, insulin activates PI3K signalling and reduces the firing rate of a proportion of SF-1^VMH^ neurons through K_ATP_ channel activation^26^. Mice lacking the IR on these subsets of neurons are partially protected from diet-induced obesity upon HFD feeding, associated with reduced systemic insulin levels and improved glucose metabolism^26^. Thus, the hyperinsulinemia present under prolonged HFD feeding predictably silences the SF-1 neurons, and IR signalling via the PI3K pathway in SF-1^VMH^ neurons mediates systemic insulin resistance and obesity in response to a HFD. Thus, the manifestation of selective insulin resistance clearly necessitates work on the underlying molecular mechanisms. Future studies should focus on region-specific mechanisms of selective hormone resistance, and, ultimately, to develop cell-specific insulin (de)sensitizers in the treatment of obesity-associated alterations such as uncontrolled HGP.

### Central insulin action controls HGP

Chronically elevated HGP contributes significantly to the hyperglycaemia associated with T2D (ref. 51). Understanding how the liver fails to respond to insulin and to the efferent signals originating from the CNS in the regulation of this process is thus of great importance.

Pharmacological approaches were the first to document a role for central insulin signalling in the control of peripheral glucose homeostasis, as infusion of insulin into the cerebral ventricle adjacent to the hypothalamus suppresses HGP and lowers blood glucose^52^. Similarly, genetic inactivation of the IR in neurons and astroglia throughout the brain causes uncontrolled HGP^53^, although this approach did not clarify which specific neuronal population(s) or other cell types in the CNS are responsible for mediating insulin's ability to inhibit HGP. A key observation in the search for the neuronal substrate explaining how brain IR signalling can inhibit HGP came from mice genetically modified to lack the IR specifically in AgRP neurons. Here, Könner *et al*. observed that failure to activate IR signalling in AgRP neurons substantially reduced the ability of peripherally applied insulin to suppress HGP under a euglycemic-hyperinsulinemic clamp. These findings thus demonstrated that the site for central insulin signalling to inhibit HGP is, indeed, AgRP neurons^13^. In agreement with these data, selective restoration of the IR specifically in AgRP neurons in addition to liver and pancreatic β-cells rescues the ability of insulin to curb HGP, whereas selective re-expression of the IR to POMC neurons in otherwise IR-deficient mice exacerbates insulin resistance and increases HGP^54^. Thus, these findings suggest a functional dichotomy in regulation of HGP originating from POMC and AgRP neurons, similar to their opposing effect on feeding and energy expenditure^19^ (Box 2).

In addition, hypothalamic insulin action reduces the breakdown of lipids (lipolysis) and promotes fatty acid and triglyceride synthesis (lipogenesis) in adipocytes through a reduction in the sympathetic tone to white adipose tissue^55^. Thus, in addition to insulin's direct effect on adipocytes and insulin action in the ARH to inhibit HGP, suppression of lipolysis as a result of insulin signalling in the brain may represent an additional mechanism for central control of glucose metabolism, since inhibition of lipolysis limits the supply of glycerol and non-esterified fatty acids from white fat, which serve as substrates for HGP.

The vagus nerve (the tenth cranial nerve) innervates large parts of the viscera and has been suggested to create the critical interface between the brain and the liver (Fig. 3). The vagus nerve also links brain IR signalling to gluconeogenesis, as central insulin action requires intact hepatic vagal nerve branches to suppress HGP^6^,^45^. Insulin hyperpolarizes AgRP neurons and inhibits their firing frequency through opening of K_ATP_ channels^13^. The reduced activity of AgRP neurons, in turn, results in IL-6-mediated activation of STAT3 signalling in the liver, and downregulates the abundance of key gluconeogenic genes, including *Pepck* and *G6Pase*^13^,^45^,^53^,^56^,^57^. Inhibition of hypothalamic PI3 kinase attenuates insulin's effect on blood glucose levels, whereas potentiating PI3K activity in the hypothalamus enhances the response to insulin in severely diabetic rats, suggesting that hypothalamic IR and PI3K signalling modulates the insulin response in diabetes^58^. Conversely, inhibition of IR signalling, for example owing to overactivation of p70S6/S6K1 kinase, in the ARH promotes hepatic insulin resistance in normal chow-fed mice, similar to what is observed in acutely HFD-fed mice^59^. These data suggest that diet-induced obesity blunts hypothalamic IR signalling and inhibits its control of HGP, substantiating a role for central insulin resistance in obese, diabetic animals. S6K1 signalling in POMC neurons is, however, also reported to suppress HGP in hyperinsulinemic clamps^60^. The disparate outcome from these experiments may not be mutually exclusive and differences in cells targeted because of varying methodology (adenoviral-based, acute pan-neuronal overexpression versus chronic POMC cell-specific gene inactivation) are likely one explanation to these seemingly discordant findings, especially considering neuronal heterogeneity, that is, existence or different subpopulations of functionally distinct POMC neurons.

**Figure 1:**
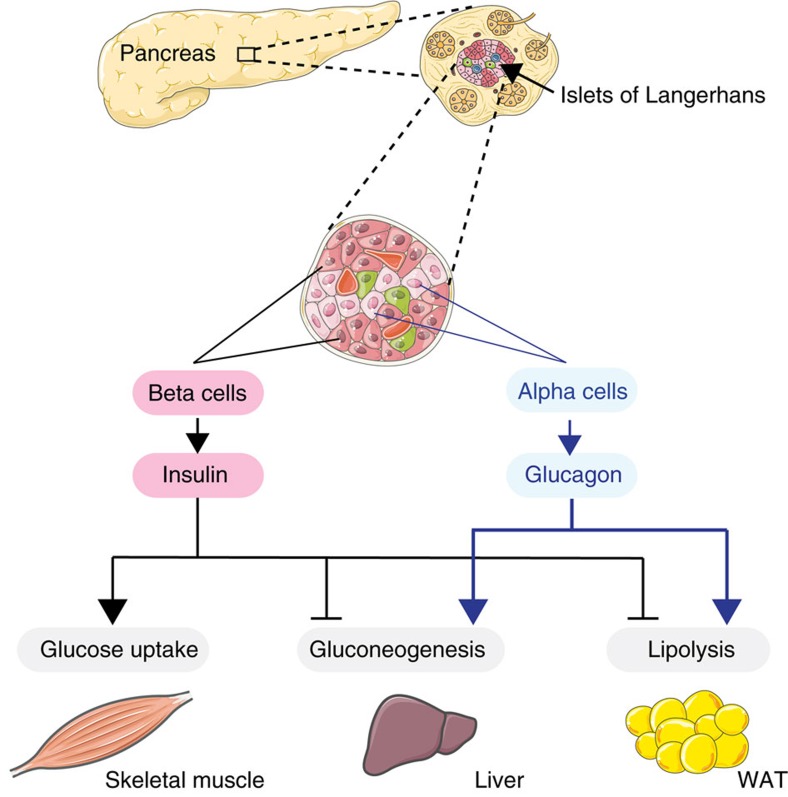
Glucoregulatory roles of the pancreatic-derived hormones insulin and glucagon. The pancreatic islets of Langherhans, containing alpha cells and beta cells, secrete glucagon and insulin respectively. Insulin and glucagon exert antagonistic effects on peripheral organs to control blood glucose levels. Insulin exerts its glucose lowering effects by stimulating glucose uptake in skeletal muscle, through inhibiting hepatic glucose production and by blunting lipolysis. By contrast, glucagon raises circulating glucose levels by increasing gluconeogenesis and lipolysis.

Insulin is not the only hormone that affects systemic glucose homeostasis through CNS-mediated mechanisms. For example, glucagon-like peptide 1 (GLP-1) augments glucose-stimulated insulin secretion and reduces HGP, likely mediated by GLP-1 receptor signalling in the ARH^61^. The peptide hormone glucagon secreted from pancreatic alpha-cells (Fig. 1), which shares a common precursor peptide with GLP-1, has on the other hand lately been reported to suppress HGP. Hypothalamic glucagon receptor activation was found to inhibit HGP through a K_ATP_ channel-dependent mechanism, and the increase in HGP from raising peripheral glucagon concentrations could be abated by blocking glucagon action in the CNS^62^,^63^. These data led to the conclusion that, in contrast to its direct actions on the liver, hypothalamic glucagon signalling inhibits HGP^62^,^63^. This was surprising, because glucagon drives HGP by direct effects on hepatocytes (Fig. 1). That a peptide promotes HGP through its stimulatory effects on the liver, and on the other hand inhibits the very same process through effects on the brain may seem counter-intuitive, as these two forces are counteracting. The findings may however point to the existence of a self-regulatory feedback loop to fine-tune HGP, in which central glucagon signalling explains why the hepatic effect of high glucagon concentration on HGP is transient, tapering off within hours even during continuous glucagon infusion.

Experiments aimed at delineating the mechanisms by which glucagon inhibits HGP are necessary to provide further clarification of glucagon's action in the brain, and could also pave the way to new treatment strategies. A monomeric peptide conjugate between glucagon, GLP-1 and GIP (glucose-dependent insulinotropic polypeptide) that acts as an agonist at each receptor vastly improves metabolic and glycemic control in obese and diabetic rodents^64^. As judged from its impact on whole-animal physiology (increased energy expenditure, reduced caloric intake and better glycemic control), it is reasonable to believe that the triple agonist exerts some of its key functions by acting on the brain. Whether the metabolic outcome of such triagonist treatment is attributed to signalling effects in the CNS, and whether activation of glucagon signalling in the hypothalamus by the polyagonist counteracts glucagon's peripheral effects on HGP are issues that merit additional studies.

Finally, whether the data in rodents on central glucagon action, with the purpose of limiting its own effects on the liver, extend to humans is important to investigate.

### Of mice and men: IR signalling in the human brain

Whether insulin action in the CNS is relevant for day-to-day or acute control of blood glucose in humans has been a matter of intense discussions^65^. While causally proving the existence of a CNS-dependent mechanism of insulin action to inhibit HGP in humans is inherently challenging, administering insulin through a spray formulation into the nose has shed some light on the physiological relevance of insulin signalling in the human brain. Intranasal application of insulin rapidly elevates levels of the hormone in the cerebrospinal fluid at concentrations that are too low to be detected in the blood, suggesting that insulin penetrated directly into the brain from the nose without increasing insulin levels in the systemic circulation^66^. Daily intranasal insulin administration over 8 weeks reduces body fat and weight in healthy men (but not woman) ranging between 0.5 and 2 kg, and acute intranasal insulin application enhances the metabolic rate to food intake by almost 20% (one treatment session; 160 IU insulin), suggesting that activation of brain IR signalling regulates peripheral fat mobilization and contributes to diet-induced thermogenesis^67^,^68^. Importantly, Heni *et al*.^69^ tested whether intranasal insulin delivery affects systemic glucose metabolism, and found evidence that reinforces a role for brain insulin action on insulin sensitivity. In their study, lean individuals required more glucose to maintain euglycemia after intranasal delivery of insulin in a clamp setting compared with placebo-treated individuals in the presence of similar venous insulin levels. These data indicated improvements in whole-body insulin sensitivity, and the amount of glucose infused interestingly correlated with increased hypothalamic activity and indices of increased parasympathetic descending vagal nerve activity^69^. Therefore, the authors concluded that short-term insulin action as a result of intranasal application of insulin improves systemic insulin sensitivity in humans, possibly via a hypothalamic-mediated vagal mechanism like in rodents^6^,^45^. However, these studies do not provide definitive evidence that endogenously produced insulin has a similar physiological role in the human brain. The responses to intranasal insulin therapy, and the cortical response to systemic hyperinsulinemia are weaker in obese humans, suggesting that obesity renders the brain less responsive to insulin^69^,^70^. This phenomenon also occurs in animals with reduced amounts of IR protein in the ARH, a situation that is accompanied by a failure to efficiently suppress HGP and whole-body insulin resistance^71^. Besides being a methodological bedrock for experiments aiming to elucidate the role of insulin signalling in the brain, the question is whether nasal insulin administration therefore represents an attractive alternative medical regimen to current therapies to treat obesity-associated diabetes.

### Central leptin signalling and systemic glucose metabolism

The brain's capacity to affect glucose homeostasis also involves mechanisms that are not exclusively dependent on insulin action. The development of T2D can be preceded by defects in not only insulin-dependent but also in insulin-independent glucose uptake more than a decade before the disease is diagnosed^72^. Thus, how efficiently glucose promotes its own disposal unrelated to insulin action predicts the future risk of developing glucose intolerance. Secreted from white adipose tissue in proportion to fat mass, leptin is intimately linked to CNS-dependent control of glucose homeostasis; as such leptin administration has been reported to rescue insulin-deficient diabetes^73^. The complete mechanism behind this observation is unclear, but leptin infusion directly into the cerebral ventricles, at a dose and route that is believed to act locally in the brain and not outside the CNS, attenuates hyperglycaemia in rats with profound diabetes due to severe insulin deficiency; this effect was independent from leptin's effect on feeding and hepatic insulin sensitivity, but involved reduced HGP and increasing glucose uptake into brain, muscle and brown fat^74^. Thus, leptin receptor signalling in the brain appears to normalize diabetic hyperglycaemia across different tissues and mechanisms, giving rise to the idea that leptin compensates for the lack of insulin in animal models of diabetes where loss of islet β-cell function is prominent^75^. In addition, combined leptin and insulin signalling in POMC neurons is broadly accepted to regulate peripheral glucose metabolism. Supporting this notion, mice lacking both the insulin and leptin receptors on POMC neurons do not suppress HGP normally, an effect associated with systemic glucose intolerance and insulin resistance^12^. Reconstitution of leptin receptor signalling on the same neurons conversely normalizes blood glucose and increases hepatic insulin sensitivity^76^. Collectively, these data point to a key role for leptin action in the ARH. However, hypoinsulinaemia as a consequence of islet failure does not seem to increase compensatory leptin receptor signalling in the CNS with the purpose of rescuing euglycemia as the hyperglycaemia usually persists in conditions characterized by insulin deficiency. Whether leptin alone can replace or compensate for insulin deficiency can thus be debated.

## Central control of pancreatic islet function

**Figure 2:**
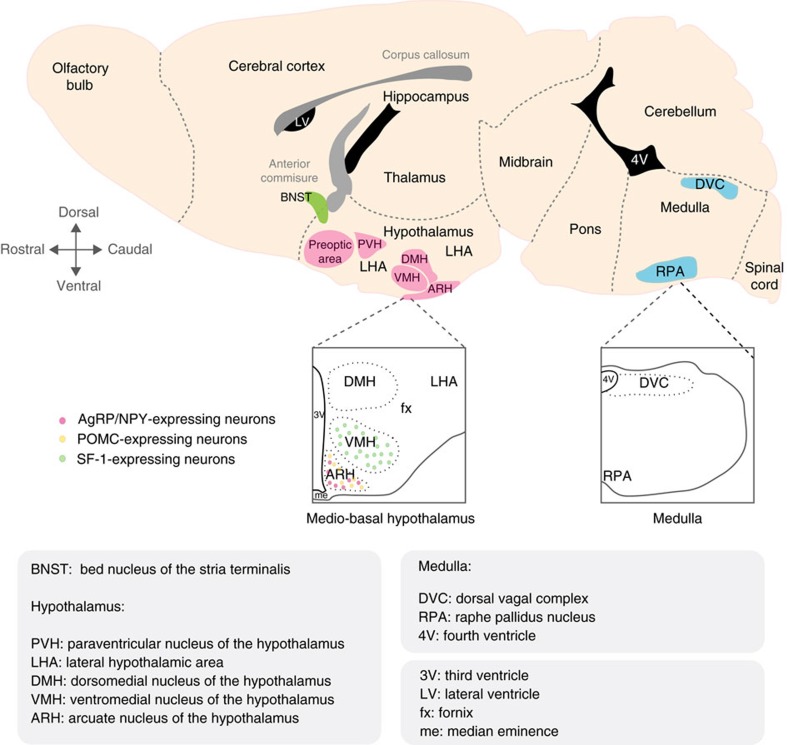
Key brain nuclei and areas involved in CNS control of glucose homeostasis. Schematic representing a sagittal section of a mouse brain in which critical brain regions controlling glucose homeostasis and peripheral insulin sensitivity as well as brown fact activity are depicted. Three main regions are highlighted: the bed nucleus of the stria terminalis (BNST), the hypothalamus and the medulla. The hypothalamus contains the preoptic area, the paraventricular nucleus (PVH), the lateral hypothalamic area (LHA), the ventromedial nucleus of the hypothalamus (VMH, where SF-1-expressing neurons reside), the dorsomedial nucleus of the hypothalamus (DMH) and the arcuate nucleus of the hypothalamus (ARH) where AgRP/NPY and POMC neurons are located. In the caudal part the brain, the medulla contains key areas such as the dorsal vagal complex (DVC) and the raphe pallidus nucleus (RPA). 3V, third ventricle; 4V, fourth ventricle; fx, fornix; LV, lateral ventricle; me, median eminence.

**Figure 3:**
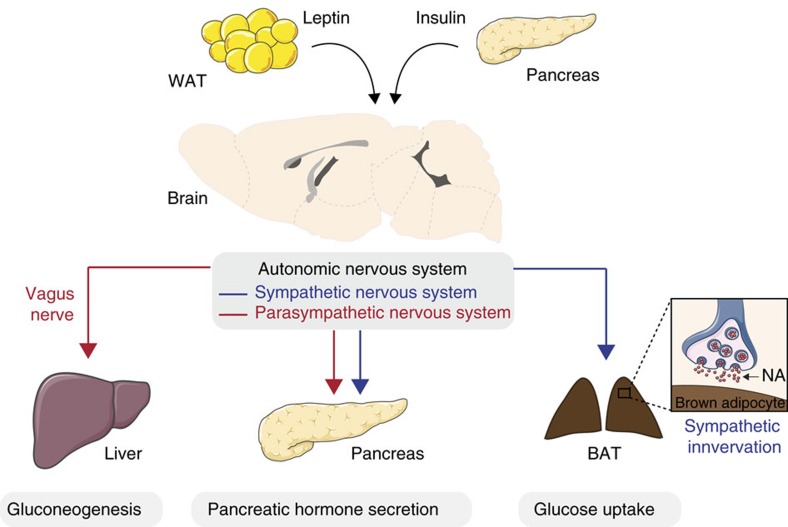
Pathways involved in the control of glucose homeostasis. The central nervous system contains high density of receptors for the white adipose tissue (WAT)-derived hormone leptin as well as receptors for the pancreatic hormone insulin. Leptin and insulin act on specific brain regions that will in turn modulate glucose utilization and production in peripheral tissue via the autonomic nervous system. Notably, the vagus nerve links brain insulin action and the liver in the control of hepatic gluconeogenesis. At the pancreatic level, the autonomic nervous system is involved in pancreatic hormone secretion. The brown adipose tissue (BAT) receives sympathetic innervation which activity directly control BAT glucose uptake. NA, noradrenaline.

The islets of the pancreas are subject to regulation by insulin signalling in the brain, and their connection with the CNS and the efferent arm of the autonomic nervous system is remarkably vulnerable during a specific developmental time window of the hypothalamic neurocircuitry^77^. Work from Vogt *et al*. has shown that feeding mothers a HFD exclusively during the lactation period leads to abnormal formation of axons from POMC neurons to the posterior part of the paraventricular nucleus of the hypothalamus (PVH (Fig. 2)), as well as of parasympathetic nerve fibres innervating the pancreas in the offspring. Ultimately, these perturbations are associated with obesity, impaired glucose-stimulated insulin secretion as well as glucose intolerance in the offspring that received fat milk^77^. On the other hand, pups genetically modified to lack the IR in POMC neurons were protected from disturbances in glucose homeostasis in response to maternal HFD feeding during lactation^77^. Thus, hyperinsulinemia may predispose the progeny of an overnutritioned breast-feeding mother for future long-lived metabolic disease through hypothalamic IR signalling, whereas the inability to sense the abnormally high levels of insulin acting on POMC neurons during lactation prevents it. Given the escalating numbers of obese and diabetic pregnant or breast-feeding women, a better understanding of metabolic, developmental programming is thus urgently needed.

Recent results obtained by combining neural tracing experiments and functional interventions directed to different hypothalamic nuclei provided new insights into the innervation of the pancreas and its influence over glucose metabolism^78^. Backtracking the CNS sites innervating the pancreas provide the evidence that glucokinase-expressing neurons in the ARH send signals via multiple synapses to this tissue^78^. Functionally, inhibiting glucose sensing in the ARH reduced insulin secretion and led to glucose intolerance, demonstrating a causal relationship between the innervation and pancreatic secretory function^78^. As the intervention was not directed towards a specific sub-set of neurons in the ARH, the identity of the neurons regulating pancreatic function remains unknown. POMC and AgRP neurons are both known to change their excitability to fluctuations in extracellular glucose concentrations in electrophysiological studies. POMC neurons are glucose excited, driven by closing of K_ATP_ channels. When POMC neurons lost the ability to sense glucose, through genetically preventing ATP-mediated closure of K_ATP_ channels, or made defective via HFD feeding, glucose tolerance is impaired^79^. Whether the effect seen stems from a failure to correctly regulate insulin secretion, however, currently remains unclear.

Other than in the ARH, *Pomc* mRNA is only expressed in the nucleus of the solitary tract within the CNS, and thus shows a very restricted expression pattern. This is in contrast to the MC4R distribution, the receptor for POMC-derived α-MSH, which is broadly expressed in the brain, including in nuclear groups in the medulla oblongata. Deletion of the MC4R in the dorsal motor nucleus of the vagus nerve (DMV), part of the dorsal vagal complex (DVC (Fig. 2))—consisting of preganglionic, parasympathetic nerve cells controlling vagal outflow—results in hyperinsulinemia and modest insulin resistance in a weight-independent manner and without changes in glucose tolerance or blood glucose levels^9^. In agreement with these findings, in obese, glucose intolerant and hyperinsulinemic MC4R-null mice, selective restoration of MC4R expression to DMV neurons attenuated the hyperinsulinemia without affecting body weight^8^. Thus, DMV MC4R signalling has an essential role in regulating blood insulin levels. Given the dissociation between improvements in insulin levels and lack of body weight reduction, these data also support the existence of divergent melanocortin pathways in control of glucose metabolism and energy balance. Whether AgRP neurons and/or other nerve cells in the melanocortin circuitry operate to regulate insulin levels, and whether the source of the ligand to the MC4R comes from the nucleus of the solitary tract or ARH, or both, should be subject of future studies. Possibly linking hypothalamic neurons to regulation of insulin secretion are insulin-sensitive GLUT-4-expressing neurons of the hypothalamus (GLUT-4^HYPO^). Cre-dependent viral tracing experiments have provided evidence that GLUT-4^HYPO^ neurons project to the DMV, and mice in which GLUT-4^HYPO^ neurons have been ablated present with elevated plasma glucose and reduced insulin levels but normal pancreatic beta-cell morphometry^80^. Accordingly, mice devoid of GLUT-4^HYPO^ neurons display impaired glucose tolerance. To that end, the authors suggested that the hyperglycaemia is a consequence of impaired insulin secretion involving a GLUT-4^HYPO^ to DMV projection^80^. While the data clearly define a role for GLUT-4^HYPO^ neurons in the control of energy and glucose metabolism, the experimental approach relied on the death of GLUT-4^HYPO^ neurons, and did not permit an evaluation on the role of GLUT-4 neurons in discrete hypothalamic areas. Genetic cell ablation may not come without caveats, such as gliosis (see below) appearing following GLUT-4^HYPO^ neuron ablation, and a vast array of neurons are GLUT-4-expressing, making the application of cell-specific excitatory or inhibitory control of viable GLUT-4^HYPO^ neurons an attractive complement for further expansion of our knowledge on their role in energy metabolism and insulin signalling^81^.

## Obesity perturbs CNS control of peripheral glucose metabolism

The reduced propensity of the CNS to respond to hormones during obesity has been extensively studied; the resistance to insulin and leptin within the melanocortin circuitry in the hypothalamus being best defined^82^,^83^,^84^. Moreover, in the CNS, activation of inflammatory processes is a key event in the manifestation of peripheral insulin resistance in obese animals^85^,^86^. Inflammatory insults to AgRP neurons have a dominant role in these processes^87^ as attenuation of the neuroinflammatory response by depriving AgRP neurons of the inhibitor of nuclear factor kappa-B kinase 2 (IKK-β) gene, an essential trigger of the immune response, protects against obesity and systemic glucose intolerance from HFD feeding^88^. Moreover, c-Jun N-terminal kinase 1- and IKK-β-dependent inflammatory signalling is sufficient to drive neuronal and systemic leptin or insulin resistance, respectively, even in the absence of HFD feeding when constitutively activated in AgRP neurons^89^. The onset of hypothalamic inflammation is rapid. Gliosis, the process of glial cells in the central nervous system reacting and proliferating to a trauma or injury (and a prominent feature of neurodegenerative diseases), surrounding AgRP neurons can be seen within three days and before fat accumulation is measurable in rodents confronted acutely to a HFD^90^. Such observations have fostered the hypothesis that neuroinflammation is an actuator of obesity development rather than a secondary consequence of weight gain. The acute HFD-induced gliosis gradually tapers off in rodents^90^,^91^, indicative of an induction of a neuroprotective mechanism, but that is eventually overridden as gliosis, leptin resistance and glucose intolerance persist upon chronic HFD feeding unless the unhealthy diet is discontinued^83^. Similar signs of inflammation have been reported in obese humans from neuroradiologic assessments of gliosis^90^, and gliosis has recently been found to associate with higher BMI, fasting insulin and HOMA-IR (Homeostatic Model Assessment, a model to assess beta-cell function and insulin resistance) in obese humans. Insulin levels and HOMA-IR did not correlate with BMI in these investigations, suggesting a link between gliosis, pancreatic responses and insulin resistance unrelated to the degree of adiposity^92^. Recent observations offer evidence in support of a neuroprotective mechanism clearly linked to inflammatory signalling, characterized by similar temporal dynamics and kinetics as the onset and disappearance of HFD-induced gliosis^93^. Here, perivascular macrophages are recruited to the blood–brain barrier of the cerebral blood vessels when the brain is challenged with a HFD to limit central inflammation. Via local vascular endothelial growth factor production and increased expression of glucose transporters (GLUT-1), these events are believed to warrant cerebral glucose homeostasis during consumption of energy-dense foods^93^.

Despite the existences of mechanisms offering acute protection of neuronal function, the extent of the exposure to fatty food is a denominator for the magnitude of hypothalamic inflammation, as prolonged HFD feeding causes leptin and insulin resistance and disturbances in peripheral glucose homeostasis. To this end, non-neuronal cells other than astrocytes and immune cells associated to the cerebral blood vessels as described above are also involved. Evidence suggests that saturated fat can be sensed predominantly by mediobasal hypothalamic, intraparenchymal microglia^94^. Activating an inflammatory M1 cytokine response to the buildup of saturated fatty acids in microglia may set the stage for hypothalamic neuronal stress and reduced leptin responsiveness, which in turn may reduce peripheral insulin sensitivity. Understanding the pathomechanisms behind diet-induced neuroinflammation is thus of high priority in the field of metabolism research, as it has implications for our understanding of obesity and insulin resistance as well as a better comprehension of the neurological complications such as neuropathies, cognitive dysfunction and stroke associated with diabetes.

## Future directions

Significant advancements to our understanding of how the brain influences peripheral glucose homeostasis have been made owing to studies revealing key brain regions and the identities of the neurons involved, their connectivity and the molecular components causally associated, as well as the peripheral organs and cellular events targeted by the brain. Specifically, HGP, brown fat glucose utilization and control of insulin secretion are processes importantly regulated by the CNS. Although great progress in this area of research has been made, several issues nonetheless remain to be resolved. To this end, while the application of techniques with high spatial resolution in neuroscientific research, relying on the existence of a known cell-specific promoter, has moved us several steps forward towards better control over functional neurocircuits, unique marker genes for many CNS cell-types potentially involved are yet nonetheless still inconspicuous. Moreover, there is extensive heterogeneity in gene expression within single CNS nuclei, and better characterization of this molecular diversity would subsequently improve our comprehension of the neuronal mechanisms controlling peripheral insulin sensitivity and glucose metabolism. Furthermore, a remaining challenge is to directly test whether processes regulating BAT activity and HGP can be exploited for the development of better and safer viable therapeutics. In fact, the beneficial effects of current anti-diabetic therapies, such as insulin supplementation, drugs triggering insulin release, insulin-resistance reducing agents and insulin-sensitizing medications are explained by peripheral actions, and although they successfully reduce hyperglycaemia, they were developed under the assumption that the brain has little, if any, influence on these processes. The inherent adverse effects including hypoglycemia, weight gain and gastrointestinal problems accompanying some of these medications are also problematic. To this end, identifying strong, selective actuators of BAT activation and agents dampening HGP will be important. Indeed, work on defining the neuronal mechanisms controlling BAT and liver biology may not only reveal potential CNS targets, but also facilitate the identification of pathways in liver and BAT directly controlled by the CNS. The findings revealing a role for myostatin signalling to impede insulin action in brown adipocytes, and myostatin's effects on reducing BAT glucose uptake in response to increased activity of AgRP neurons, is one example of such synergistic development. Key players of energy and glucose homeostasis, including AgRP neurons, myostatin and melanocortin's, are found in both rodents and humans, and pharmacological myostatin inhibition improves insulin sensitivity^14^,^95^. Realistically, drug candidates in the myostatin signalling cascade, well-studied in the context of muscle growth, sarcopenia and cachexia, could rapidly be advanced into clinical trials assessing their therapeutic potential to moderate insulin resistance.

There is also a need to define novel regulators of key glucoregulatory neuronal populations, which may lead to innovative therapies. For instance, recent publications identified the purinergic-receptor 6 (P2Y6) as novel regulator of AgRP neuron activity and further revealed that selectively abrogating P2Y6 signalling in AgRP neurons alleviates obesity-associated insulin resistance^96^. Translational studies will be necessary to validate if P2Y6-antagonism represents a pharmaceutical way for diabetic treatment. Finally, as failure to suppress HGP or impaired insulin sensitivity and glucose intolerance may develop as consequences of central hormone resistance, especially upon central inflammation, continued efforts in defining the intracellular pathways that are altered in obesity are required, and whether normalization of their function rescues energy and glucose metabolism. Ideally, this knowledge will facilitate to the development of novel pharmaceutical interventions for the treatment of obesity and diabetes. Such discoveries are also expected to furnish our understanding of neuronal control mechanisms of whole-body insulin sensitivity and glucose metabolism.

## Additional information

**How to cite this article:** Ruud, J. *et al*. Neuronal control of peripheral insulin sensitivity and glucose metabolism. *Nat. Commun.* **8**, 15259 doi: 10.1038/ncomms15259 (2017).

**Publisher's note:** Springer Nature remains neutral with regard to jurisdictional claims in published maps and institutional affiliations.

## Figures and Tables

**Table 1:** Cell type- and neurocircuit-specific manipulations reported to modulate peripheral insulin sensitivity and/or glucose homeostasis acutely or chronically.

**Region/nucleus**	**Target/neuron studied**	**Method/neuronal activity**	**Time scale**	**Observed outcome**	**Peripheral effector(s)**	**Ref.**
ARH	ARH^POMC^	Chemogenetics (hM3D_Gq_, ↑)	Acute	Insulin sensitivity ↔Glucose tolerance ↔	—	14
	ARH^AgRP^	Chemogenetics (hM3D_Gq_, ↑)	Acute	Insulin sensitivity ↓Glucose tolerance ↓	BAT glucose uptake ↓	14
	ARH^AgRP^	Optogenetics (ChR2, ↑)Somatic photostimulation	Acute	Insulin sensitivity ↓	BAT (*myostatin* mRNA ↑)	14
	AgRP^IR^	Conditional deletion of the IR in AgRP neurons	Chronic	Systemic insulin sensitivity ↔	HGP ↑	13
	AgRP^IR^	Selective re-expression of the IR in AgRP neurons	Chronic	Systemic insulin sensitivity ↔	HGP ↓	54
	POMC^IR^	Selective re-expression of the IR in POMC neurons	Chronic	Insulin sensitivity ↓	HGP ↑	54
	POMC^Kir6.2^	Conditional deletion of Kir6.2 in POMC neurons	Chronic	Glucose tolerance ↓	?	79
	POMC^IR, LepR^	Conditional deletion of the IR and the LepR in POMC neurons	Chronic	Insulin sensitivity ↓Glucose tolerance ↓	HGP ↑	12
	POMC^LepR^	Selective re-expression of the LepR in POMC neurons	Chronic	Insulin sensitivity↑	HGP ↓	76
VMH	VMH^SF-1^	Optogenetics (SwiChR_CA_, ↓)	Acute	Response to hypoglycemia ↓	Abnormal CRR	27
	VMH^SF-1^	Optogenetics (ChR2, ↑)Somatic photostimulation	Acute	Blood glucose levels↑	HGP ↑Corticosterone ↑Glucagon ↑	27
	VMH^Glucokinase^	Electromagnetics (↑)	Acute	Blood glucose levels ↑	HGP ↑	28
	VMH^Glucokinase^	Electromagnetics (↓)	Acute	Blood glucose levels ↓	HGP ↓	28
	SF-1^IR^	Conditional deletion of the IR in SF-1 neurons (HFD)	Chronic	Glucose tolerance ↑Insulin sensitivity ↑	?	26
BNST	ARH^AgRP^→aBNST_vl	Optogenetics (ChR2, ↑)	Acute	Insulin sensitivity ↓	BAT (*myostatin* mRNA ↑)	14
	VMH^SF-1^→aBNST	Optogenetics (ChR2, ↑)	Acute	Blood glucose levels ↑	Corticosterone ↑Glucagon ↑	27
LHA	ARH^AgRP^→LHA	Optogenetics (ChR2, ↑)	Acute	Insulin sensitivity ↓	?	14
	LHA^MC4R^	Selective MC4R re-expression in LHA neurons of MC4R-null mice	Chronic	Hyperglycemia ↓Glucose tolerance ↑	BAT glucose uptake ↑	35
DMV	Phox2b^MC4R^	Conditional deletion of MC4R	Chronic	Insulin sensitivity ↓	?	9
	DMV^MC4R^	Selective MC4R re-expression in MC4R-null mice	Chronic	Hyperinsulinemia ↓	?	8

**Table 2:** Cell type-specific manipulations reported to alter food intake.

**Region/nucleus**	**Neuronal population**	**Method**	**Change in neuronal activity, via receptor or ion-channel**	**Food intake**	**Ref.**
PVH	Sim1	Chemogenetics	↓, hM4D	↑	10
	TRH	Chemogenetics	↑, hM3D	↑	97
	PACAP	Chemogenetics	↑, hM3D	↑	97
ARH	AgRP	Optogenetics	↑, ChR2	↑	19
	AgRP	Chemogenetics	↑, hM3D	↑	16
	AgRP	Chemogenetics	↓, hM4D	↓	16
	POMC	Optogenetics	↑, ChR2	↓	19
	POMC	Chemogenetics	↑, hM3D	↓	98
	POMC	Chemogenetics	↓, hM4D	↑	10
LHA	VGLUT2	Optogenetics	↑, ChR2	↓	34
DVC	POMC	Chemogenetics	↑, hM3D	↓	98

## Claude Bernard revisited.

In the 1850s, physiologist Claude Bernard observed that manipulation to the floor of the fourth ventricle in the hindbrain of experimental animals caused blood glucose levels to rise above normal, and that the excess sugar was excreted in the urine^99^. Befittingly, Bernard is associated with articulating the concept of homeostasis (*milieu intérieur*; French for ‘the environment within'). Walter Bradford Cannon later conceptualized and developed it further. Although these observations and Bernard's finding that the liver contains glycogen (indicating that the liver stores an energy source that can be converted into glucose) were epoch-making, the extraordinary important discovery of insulin in the 1920s by Banting and fellow workers made scientists to shift their focus and research activities. With diminished enthusiasm for the brain as an interesting target for intervention, research was now devoted to deciphering insulin action in peripheral organs and defects in pancreatic insulin secretion. In hindsight, however, and considering that the brain governs control of most homeostatic networks, it seems improbable that glucose metabolism would be controlled by mechanisms independent of the CNS.

## AgRP and POMC ARH neurons are key regulators of food intake.

The activity of POMC and AgRP neurons bi-modally and rapidly controls appetitive behavior even upon mere sensory perception of food^100^. Activation of POMC^ARH^ neurons selectively suppresses appetite^19^,^98^ (Table 2), and mutations in the POMC gene are associated with obesity in a range of species including humans, mice and dogs^101^. Best known for signaling satiety, recent intriguing data reveal a previously unprecedented function of a subset of POMC neurons to promote feeding behavior through cannabinoid-receptor mediated release of β-endorphin, an endogenous opioid neuropeptide originating from the POMC precursor molecule^102^. By contrast, AgRP neurons are hunger sensitive and signal energy deficits: their activation rapidly evokes eating and their ablation in the adult animal causes rapid weight loss due to cessation of feeding^16^,^19^,^103^,^104^. Mechanistically, the neuropeptide AgRP competes with α-melanocyte-stimulating hormone (α-MSH) released from POMC neurons for binding sites on the melanocortin 4-receptor (MC4R), blocks the coupling to a G_αs_ signaling pathway, and promotes feeding when AgRP has the upper hand. Besides the canonical view of neuronal MC4R signaling, new fascinating data however suggest that AgRP can act independently of G_αs_ and through regulating the pore state of an inwardly rectifying potassium channel, Kir7.1. According to these results, binding of AgRP onto MC4R opens Kir7.1 and hyperpolarizes the downstream target second order neuron without its inhibition of α-MSH binding, some of which are located in the paraventricular hypothalamus, a nucleus crucial to the control of hunger (Fig. 2)^97^,^105^.

## References

[b1] Ng M. . Global, regional, and national prevalence of overweight and obesity in children and adults during 1980-2013: a systematic analysis for the Global Burden of Disease Study 2013. Lancet 384, 766–781 (2014). 24880830 10.1016/S0140-6736(14)60460-8 PMC4624264

[b2] Wild S., Roglic G., Green A., Sicree R. & King H. Global prevalence of diabetes: estimates for the year 2000 and projections for 2030. Diabetes Care 27, 1047–1053 (2004). 15111519 10.2337/diacare.27.5.1047

[b3] Stevens J. . The effect of age on the association between body-mass index and mortality. N. Engl. J. Med. 338, 1–7 (1998). 9414324 10.1056/NEJM199801013380101

[b4] Ezzati M., Lopez A. D., Rodgers A., Vander Hoorn S. & Murray C. J. Comparative Risk Assessment Collaborating G. Selected major risk factors and global and regional burden of disease. Lancet 360, 1347–1360 (2002). 12423980 10.1016/S0140-6736(02)11403-6

[b5] Kasuga M., Karlsson F. A. & Kahn C. R. Insulin stimulates the phosphorylation of the 95,000-dalton subunit of its own receptor. Science 215, 185–187 (1982). 7031900 10.1126/science.7031900

[b6] Pocai A., Obici S., Schwartz G. J. & Rossetti L. A brain-liver circuit regulates glucose homeostasis. Cell Metab. 1, 53–61 (2005). 16054044 10.1016/j.cmet.2004.11.001

[b7] Filippi B. M., Yang C. S., Tang C. & Lam T. K. Insulin activates Erk1/2 signaling in the dorsal vagal complex to inhibit glucose production. Cell Metab. 16, 500–510 (2012). 23040071 10.1016/j.cmet.2012.09.005

[b8] Rossi J. . Melanocortin-4 receptors expressed by cholinergic neurons regulate energy balance and glucose homeostasis. Cell Metab. 13, 195–204 (2011) ***One in a series of studies relying on selective MC4R restoration to gain better understanding of the defects typical of MC4R deficiency. By examining MC4R signaling in various autonomic nervous system neurons, diverging pathways mediating the effects of melanocortins on energy balance and glucose homeostasis are elegantly covered***. 21284986 10.1016/j.cmet.2011.01.010 PMC3033043

[b9] Berglund E. D. . Melanocortin 4 receptors in autonomic neurons regulate thermogenesis and glycemia. Nat. Neurosci. 17, 911–913 (2014). 24908101 10.1038/nn.3737 PMC4090093

[b10] Atasoy D., Betley J. N., Su H. H. & Sternson S. M. Deconstruction of a neural circuit for hunger. Nature 488, 172–177 (2012) ***A comprehensive article defining in detail, using circuit mapping to probe a number of postsynaptic targets of starvation-sensitive nerve cells, the functional connection downstream of AgRP neurons in evoked feeding responses. Introduced a concept by which AgRP neurons target oxytocin neurons in the PVH, and inhibit these neurons to promote feeding***. 22801496 10.1038/nature11270 PMC3416931

[b11] Stachniak T. J., Ghosh A. & Sternson S. M. Chemogenetic synaptic silencing of neural circuits localizes a hypothalamus→midbrain pathway for feeding behavior. Neuron 82, 797–808 (2014). 24768300 10.1016/j.neuron.2014.04.008 PMC4306349

[b12] Hill J. W. . Direct insulin and leptin action on pro-opiomelanocortin neurons is required for normal glucose homeostasis and fertility. Cell Metab. 11, 286–297 (2010). 20374961 10.1016/j.cmet.2010.03.002 PMC2854520

[b13] Konner A. C. . Insulin action in AgRP-expressing neurons is required for suppression of hepatic glucose production. Cell Metab. 5, 438–449 (2007) ***This work constituted the first demonstration that insulin action in the CNS controls HGP specifically via AgRP neurons***. 17550779 10.1016/j.cmet.2007.05.004

[b14] Steculorum S. M. . AgRP neurons control systemic insulin sensitivity via myostatin expression in brown adipose tissue. Cell 165, 125–138 (2016) ***Via a distinct and overlapping functional architecture of neurocircuits, this paper explains how AgRP neuron activation acutely impairs insulin sensitivity. It documented for the first time that AgRP neurons rapidly re-program BAT gene expression; a switch towards a myogenic gene profile was seen upon activation of these neurons***. 27015310 10.1016/j.cell.2016.02.044 PMC5157157

[b15] Guo T. . Myostatin inhibition in muscle, but not adipose tissue, decreases fat mass and improves insulin sensitivity. PLoS ONE 4, e4937 (2009). 19295913 10.1371/journal.pone.0004937 PMC2654157

[b16] Krashes M. J. . Rapid, reversible activation of AgRP neurons drives feeding behavior in mice. J. Clin. Invest. 121, 1424–1428 (2011) ***Much-quoted paper, and one of the first reported achievements on the use of DREADDs expressed in neurons previously implicated in energy homeostasis: stimulation of AgRP neurons made mice eat more in a matter of minutes, whereas inhibition reduced feeding in hungry mice***. 21364278 10.1172/JCI46229 PMC3069789

[b17] Joly-Amado A. . Hypothalamic AgRP-neurons control peripheral substrate utilization and nutrient partitioning. EMBO J. 31, 4276–4288 (2012). 22990237 10.1038/emboj.2012.250 PMC3501217

[b18] Betley J. N., Cao Z. F., Ritola K. D. & Sternson S. M. Parallel, redundant circuit organization for homeostatic control of feeding behavior. Cell 155, 1337–1350 (2013) ***An elegant paper based on cell-type-specific circuit manipulation and projection-specific anatomical analysis, revealing that stimulation of AgRP neuron projections in numerous brain areas elicits feeding behaviour. Although AgRP neurons project broadly throughout the brain, they appear to project primarily in a one-to-one configuration, and each projection site received innervation from a distinct subgroup of AgRP neurons capable of controlling food intake alone***. 24315102 10.1016/j.cell.2013.11.002 PMC3970718

[b19] Aponte Y., Atasoy D. & Sternson S. M. AGRP neurons are sufficient to orchestrate feeding behavior rapidly and without training. Nat. Neurosci. 14, 351–355 (2011). 21209617 10.1038/nn.2739 PMC3049940

[b20] Wu Q., Howell M. P., Cowley M. A. & Palmiter R. D. Starvation after AgRP neuron ablation is independent of melanocortin signaling. Proc. Natl Acad. Sci. USA 105, 2687–2692 (2008). 18272480 10.1073/pnas.0712062105 PMC2268197

[b21] Nakajima K. . Gs-coupled GPCR signalling in AgRP neurons triggers sustained increase in food intake. Nat. Commun. 7, 10268 (2016). 26743492 10.1038/ncomms10268 PMC4729878

[b22] Krashes M. J., Shah B. P., Koda S. & Lowell B. B. Rapid versus delayed stimulation of feeding by the endogenously released AgRP neuron mediators GABA, NPY, and AgRP. Cell Metab. 18, 588–595 (2013). 24093681 10.1016/j.cmet.2013.09.009 PMC3822903

[b23] Shi Y. C. . Arcuate NPY controls sympathetic output and BAT function via a relay of tyrosine hydroxylase neurons in the PVN. Cell Metab. 17, 236–248 (2013). 23395170 10.1016/j.cmet.2013.01.006

[b24] Fenselau H. . A rapidly acting glutamatergic ARC→PVH satiety circuit postsynaptically regulated by alpha-MSH. Nat. Neurosci. 20, 42–51 (2017). 27869800 10.1038/nn.4442 PMC5191921

[b25] Shimazu T., Fukuda A. & Ban T. Reciprocal influences of the ventromedial and lateral hypothalamic nuclei on blood glucose level and liver glycogen content. Nature 210, 1178–1179 (1966). 5964188 10.1038/2101178a0

[b26] Klockener T. . High-fat feeding promotes obesity via insulin receptor/PI3K-dependent inhibition of SF-1 VMH neurons. Nat. Neurosci. 14, 911–918 (2011). 21642975 10.1038/nn.2847 PMC3371271

[b27] Meek T. H. . Functional identification of a neurocircuit regulating blood glucose. Proc. Natl Acad. Sci. USA 113, E2073–E2082 (2016) ***A comprehensive article that covers both connectivity and functional aspects, with particular attention to a subset of VMH neurons in glucose counter-regulation. The authors identify an activating projection from the VMH to the aBNST that increases blood glucose levels; silencing the VMH neurons impaired normalization of blood glucose levels during hypoglycemia***. 27001850 10.1073/pnas.1521160113 PMC4833243

[b28] Stanley S. A. . Bidirectional electromagnetic control of the hypothalamus regulates feeding and metabolism. Nature 531, 647–650 (2016). 27007848 10.1038/nature17183 PMC4894494

[b29] Meister M. Physical limits to magnetogenetics. Elife 5, e17210 (2016). 27529126 10.7554/eLife.17210 PMC5016093

[b30] Bartelt A. . Brown adipose tissue activity controls triglyceride clearance. Nat. Med. 17, 200–205 (2011). 21258337 10.1038/nm.2297

[b31] Yu S. . Glutamatergic preoptic area neurons that express leptin receptors drive temperature-dependent body weight homeostasis. J. Neurosci. 36, 5034–5046 (2016). 27147656 10.1523/JNEUROSCI.0213-16.2016 PMC4854966

[b32] Nakamura K. & Morrison S. F. A thermosensory pathway that controls body temperature. Nat. Neurosci. 11, 62–71 (2008). 18084288 10.1038/nn2027 PMC2423341

[b33] Lazarus M. . EP3 prostaglandin receptors in the median preoptic nucleus are critical for fever responses. Nat. Neurosci. 10, 1131–1133 (2007). 17676060 10.1038/nn1949

[b34] Jennings J. H., Rizzi G., Stamatakis A. M., Ung R. L. & Stuber G. D. The inhibitory circuit architecture of the lateral hypothalamus orchestrates feeding. Science 341, 1517–1521 (2013). 24072922 10.1126/science.1241812 PMC4131546

[b35] Morgan D. A. . Regulation of glucose tolerance and sympathetic activity by MC4R signaling in the lateral hypothalamus. Diabetes 64, 1976–1987 (2015) ***A paper offering shedding light on the complicated topic of melanocortin signaling. Discrete MC4R restoration in the LHA was found to reduce glucose intolerance in otherwise whole-body MC4R-deficient mice; the improvement could be linked to sympathetic nervous system-dependent control of BAT glucose utilization, occurring without changes in body weight***. 25605803 10.2337/db14-1257 PMC4439564

[b36] Cypess A. M. . Identification and importance of brown adipose tissue in adult humans. N. Engl. J. Med. 360, 1509–1517 (2009) ***Provided convincing experimental evidence for the (re)discovery of functionally active BAT in adult humans. Such data were independently described in similarly classic papers the same year in references 37–39, work that revitalized the field of brown fat research and fuelled interest in BAT glucoregulatory properties***. 19357406 10.1056/NEJMoa0810780 PMC2859951

[b37] van Marken Lichtenbelt W. D. . Cold-activated brown adipose tissue in healthy men. N. Engl. J. Med. 360, 1500–1508 (2009). 19357405 10.1056/NEJMoa0808718

[b38] Virtanen K. A. . Functional brown adipose tissue in healthy adults. N. Engl. J. Med. 360, 1518–1525 (2009). 19357407 10.1056/NEJMoa0808949

[b39] Saito M. . High incidence of metabolically active brown adipose tissue in healthy adult humans: effects of cold exposure and adiposity. Diabetes 58, 1526–1531 (2009). 19401428 10.2337/db09-0530 PMC2699872

[b40] Orava J. . Different metabolic responses of human brown adipose tissue to activation by cold and insulin. Cell Metab. 14, 272–279 (2011). 21803297 10.1016/j.cmet.2011.06.012

[b41] Ouellet V. . Outdoor temperature, age, sex, body mass index, and diabetic status determine the prevalence, mass, and glucose-uptake activity of 18F-FDG-detected BAT in humans. J. Clin. Endocrinol. Metab. 96, 192–199 (2011). 20943785 10.1210/jc.2010-0989

[b42] Chondronikola M. . Brown adipose tissue improves whole-body glucose homeostasis and insulin sensitivity in humans. Diabetes 63, 4089–4099 (2014). 25056438 10.2337/db14-0746 PMC4238005

[b43] Lee P. . Brown adipose tissue exhibits a glucose-responsive thermogenic biorhythm in humans. Cell Metab. 23, 602–609 (2016). 26972823 10.1016/j.cmet.2016.02.007

[b44] Gerhart-Hines Z. . The nuclear receptor Rev-erbalpha controls circadian thermogenic plasticity. Nature 503, 410–413 (2013). 24162845 10.1038/nature12642 PMC3839416

[b45] Pocai A. . Hypothalamic K(ATP) channels control hepatic glucose production. Nature 434, 1026–1031 (2005). 15846348 10.1038/nature03439

[b46] Bruning J. C. . Role of brain insulin receptor in control of body weight and reproduction. Science 289, 2122–2125 (2000) ***This paper represents some of the first genetic evidence for a new key site for insulin to affect energy disposal and fuel metabolism, the brain. In the advent of conditional mutagenesis, deletion of the IR in brain neurons and astroglia recapitulated several key features of the metabolic syndrome***. 11000114 10.1126/science.289.5487.2122

[b47] Kishore P. . Activation of K(ATP) channels suppresses glucose production in humans. J. Clin. Invest. 121, 4916–4920 (2011). 22056385 10.1172/JCI58035 PMC3225998

[b48] Spanswick D., Smith M. A., Mirshamsi S., Routh V. H. & Ashford M. L. Insulin activates ATP-sensitive K+ channels in hypothalamic neurons of lean, but not obese rats. Nat. Neurosci. 3, 757–758 (2000). 10903566 10.1038/77660

[b49] Qiu J. . Insulin excites anorexigenic proopiomelanocortin neurons via activation of canonical transient receptor potential channels. Cell Metab. 19, 682–693 (2014). 24703699 10.1016/j.cmet.2014.03.004 PMC4183666

[b50] Hausen A. C. . Insulin-dependent activation of MCH neurons impairs locomotor activity and insulin sensitivity in obesity. Cell Rep. 17, 2512–2521 (2016). 27926856 10.1016/j.celrep.2016.11.030

[b51] Consoli A., Nurjhan N., Capani F. & Gerich J. Predominant role of gluconeogenesis in increased hepatic glucose production in NIDDM. Diabetes 38, 550–557 (1989). 2653926 10.2337/diab.38.5.550

[b52] Obici S., Zhang B. B., Karkanias G. & Rossetti L. Hypothalamic insulin signaling is required for inhibition of glucose production. Nat. Med. 8, 1376–1382 (2002). 12426561 10.1038/nm1202-798

[b53] Inoue H. . Role of hepatic STAT3 in brain-insulin action on hepatic glucose production. Cell Metab. 3, 267–275 (2006). 16581004 10.1016/j.cmet.2006.02.009

[b54] Lin H. V. . Divergent regulation of energy expenditure and hepatic glucose production by insulin receptor in agouti-related protein and POMC neurons. Diabetes 59, 337–346 (2010). 19933998 10.2337/db09-1303 PMC2809966

[b55] Scherer T. . Brain insulin controls adipose tissue lipolysis and lipogenesis. Cell Metab. 13, 183–194 (2011). 21284985 10.1016/j.cmet.2011.01.008 PMC3061443

[b56] Koch L. . Central insulin action regulates peripheral glucose and fat metabolism in mice. J. Clin. Invest. 118, 2132–2147 (2008). 18451994 10.1172/JCI31073 PMC2350427

[b57] Wunderlich F. T. . Interleukin-6 signaling in liver-parenchymal cells suppresses hepatic inflammation and improves systemic insulin action. Cell Metab. 12, 237–249 (2010). 20816090 10.1016/j.cmet.2010.06.011

[b58] Gelling R. W. . Insulin action in the brain contributes to glucose lowering during insulin treatment of diabetes. Cell Metab. 3, 67–73 (2006). 16399506 10.1016/j.cmet.2005.11.013

[b59] Ono H. . Activation of hypothalamic S6 kinase mediates diet-induced hepatic insulin resistance in rats. J. Clin. Invest. 118, 2959–2968 (2008). 18618016 10.1172/JCI34277 PMC2447927

[b60] Smith M. A. . Ribosomal S6K1 in POMC and AgRP neurons regulates glucose homeostasis but not feeding behavior in mice. Cell Rep. 11, 335–343 (2015). 25865886 10.1016/j.celrep.2015.03.029 PMC4410943

[b61] Sandoval D. A., Bagnol D., Woods S. C., D'Alessio D. A. & Seeley R. J. Arcuate glucagon-like peptide 1 receptors regulate glucose homeostasis but not food intake. Diabetes 57, 2046–2054 (2008). 18487451 10.2337/db07-1824 PMC2494674

[b62] Mighiu P. I. . Hypothalamic glucagon signaling inhibits hepatic glucose production. Nat. Med. 19, 766–772 (2013). 23685839 10.1038/nm.3115

[b63] Abraham M. A. . Hypothalamic glucagon signals through the KATP channels to regulate glucose production. Mol. Metab. 3, 202–208 (2014). 24634823 10.1016/j.molmet.2013.11.007 PMC3953686

[b64] Finan B. . A rationally designed monomeric peptide triagonist corrects obesity and diabetes in rodents. Nat. Med. 21, 27–36 (2015). 25485909 10.1038/nm.3761

[b65] Edgerton D. S. & Cherrington A. D. Is brain insulin action relevant to the control of plasma glucose in humans? Diabetes 64, 696–699 (2015). 25713193 10.2337/db14-1666 PMC4876740

[b66] Born J. . Sniffing neuropeptides: a transnasal approach to the human brain. Nat. Neurosci. 5, 514–516 (2002). 11992114 10.1038/nn849

[b67] Benedict C. . Intranasal insulin enhances postprandial thermogenesis and lowers postprandial serum insulin levels in healthy men. Diabetes 60, 114–118 (2011). 20876713 10.2337/db10-0329 PMC3012162

[b68] Hallschmid M. . Intranasal insulin reduces body fat in men but not in women. Diabetes 53, 3024–3029 (2004). 15504987 10.2337/diabetes.53.11.3024

[b69] Heni M. . Central insulin administration improves whole-body insulin sensitivity via hypothalamus and parasympathetic outputs in men. Diabetes 63, 4083–4088 (2014). 25028522 10.2337/db14-0477

[b70] Tschritter O. . The cerebrocortical response to hyperinsulinemia is reduced in overweight humans: a magnetoencephalographic study. Proc. Natl Acad. Sci. USA 103, 12103–12108 (2006). 16877540 10.1073/pnas.0604404103 PMC1567704

[b71] Obici S., Feng Z., Karkanias G., Baskin D. G. & Rossetti L. Decreasing hypothalamic insulin receptors causes hyperphagia and insulin resistance in rats. Nat. Neurosci. 5, 566–572 (2002). 12021765 10.1038/nn0602-861

[b72] Martin B. C. . Role of glucose and insulin resistance in development of type 2 diabetes mellitus: results of a 25-year follow-up study. Lancet 340, 925–929 (1992). 1357346 10.1016/0140-6736(92)92814-v

[b73] Fujikawa T. . Leptin engages a hypothalamic neurocircuitry to permit survival in the absence of insulin. Cell Metab. 18, 431–444 (2013). 24011077 10.1016/j.cmet.2013.08.004 PMC3890693

[b74] German J. P. . Leptin activates a novel CNS mechanism for insulin-independent normalization of severe diabetic hyperglycemia. Endocrinology 152, 394–404 (2011). 21159853 10.1210/en.2010-0890 PMC3037161

[b75] Yu X., Park B. H., Wang M. Y., Wang Z. V. & Unger R. H. Making insulin-deficient type 1 diabetic rodents thrive without insulin. Proc. Natl Acad. Sci. USA 105, 14070–14075 (2008). 18779578 10.1073/pnas.0806993105 PMC2544580

[b76] Berglund E. D. . Direct leptin action on POMC neurons regulates glucose homeostasis and hepatic insulin sensitivity in mice. J. Clin. Invest. 122, 1000–1009 (2012). 22326958 10.1172/JCI59816 PMC3287225

[b77] Vogt M. C. . Neonatal insulin action impairs hypothalamic neurocircuit formation in response to maternal high-fat feeding. Cell 156, 495–509 (2014). 24462248 10.1016/j.cell.2014.01.008 PMC4101521

[b78] Rosario W. . The brain to pancreatic islet neuronal map reveals differential glucose regulation from distinct hypothalamic regions. Diabetes 65, 2711–2723 (2016). 27207534 10.2337/db15-0629 PMC5001176

[b79] Parton L. E. . Glucose sensing by POMC neurons regulates glucose homeostasis and is impaired in obesity. Nature 449, 228–232 (2007). 17728716 10.1038/nature06098

[b80] Ren H., Lu T. Y., McGraw T. E. & Accili D. Anorexia and impaired glucose metabolism in mice with hypothalamic ablation of Glut4 neurons. Diabetes 64, 405–417 (2015). 25187366 10.2337/db14-0752 PMC4303970

[b81] Ren H. . Glut4 expression defines an insulin-sensitive hypothalamic neuronal population. Mol. Metab. 3, 452–459 (2014). 24944904 10.1016/j.molmet.2014.04.006 PMC4060214

[b82] Munzberg H., Flier J. S. & Bjorbaek C. Region-specific leptin resistance within the hypothalamus of diet-induced obese mice. Endocrinology 145, 4880–4889 (2004). 15271881 10.1210/en.2004-0726

[b83] Enriori P. J. . Diet-induced obesity causes severe but reversible leptin resistance in arcuate melanocortin neurons. Cell Metab. 5, 181–194 (2007). 17339026 10.1016/j.cmet.2007.02.004

[b84] Kleinridders A. . MyD88 signaling in the CNS is required for development of fatty acid-induced leptin resistance and diet-induced obesity. Cell Metab. 10, 249–259 (2009). 19808018 10.1016/j.cmet.2009.08.013 PMC3898351

[b85] Belgardt B. F. . Hypothalamic and pituitary c-Jun N-terminal kinase 1 signaling coordinately regulates glucose metabolism. Proc. Natl Acad. Sci. USA 107, 6028–6033 (2010). 20231445 10.1073/pnas.1001796107 PMC2851918

[b86] Jais A. & Bruning J. C. Hypothalamic inflammation in obesity and metabolic disease. J. Clin. Invest. 127, 24–32 (2017). 28045396 10.1172/JCI88878 PMC5199695

[b87] Olofsson L. E., Unger E. K., Cheung C. C. & Xu A. W. Modulation of AgRP-neuronal function by SOCS3 as an initiating event in diet-induced hypothalamic leptin resistance. Proc. Natl Acad. Sci. USA 110, E697–E706 (2013). 23386726 10.1073/pnas.1218284110 PMC3581908

[b88] Zhang X. . Hypothalamic IKKbeta/NF-kappaB and ER stress link overnutrition to energy imbalance and obesity. Cell 135, 61–73 (2008). 18854155 10.1016/j.cell.2008.07.043 PMC2586330

[b89] Tsaousidou E. . Distinct roles for JNK and IKK activation in agouti-related peptide neurons in the development of obesity and insulin resistance. Cell Rep. 9, 1495–1506 (2014). 25456138 10.1016/j.celrep.2014.10.045

[b90] Thaler J. P. . Obesity is associated with hypothalamic injury in rodents and humans. J. Clin. Invest. 122, 153–162 (2012). 22201683 10.1172/JCI59660 PMC3248304

[b91] Berkseth K. E. . Hypothalamic gliosis associated with high-fat diet feeding is reversible in mice: a combined immunohistochemical and magnetic resonance imaging study. Endocrinology 155, 2858–2867 (2014). 24914942 10.1210/en.2014-1121 PMC4098007

[b92] Schur E. A. . Radiologic evidence that hypothalamic gliosis is associated with obesity and insulin resistance in humans. Obesity (Silver Spring) 23, 2142–2148 (2015). 26530930 10.1002/oby.21248 PMC4634110

[b93] Jais A. . Myeloid-cell-derived VEGF maintains brain glucose uptake and limits cognitive impairment in obesity. Cell 166, 1338–1340 (2016). 27565353 10.1016/j.cell.2016.08.010

[b94] Valdearcos M. . Microglia dictate the impact of saturated fat consumption on hypothalamic inflammation and neuronal function. Cell Rep. 9, 2124–2138 (2014). 25497089 10.1016/j.celrep.2014.11.018 PMC4617309

[b95] Camporez J. P. . Anti-myostatin antibody increases muscle mass and strength and improves insulin sensitivity in old mice. Proc. Natl Acad. Sci. USA 113, 2212–2217 (2016). 26858428 10.1073/pnas.1525795113 PMC4776508

[b96] Steculorum S. M. . Inhibition of P2Y6 signaling in AgRP neurons reduces food intake and improves systemic insulin sensitivity in obesity. Cell Rep. 18, 1587–1597 (2017). 28199831 10.1016/j.celrep.2017.01.047

[b97] Krashes M. J. . An excitatory paraventricular nucleus to AgRP neuron circuit that drives hunger. Nature 507, 238–242 (2014). 24487620 10.1038/nature12956 PMC3955843

[b98] Zhan C. . Acute and long-term suppression of feeding behavior by POMC neurons in the brainstem and hypothalamus, respectively. J. Neurosci. 33, 3624–3632 (2013). 23426689 10.1523/JNEUROSCI.2742-12.2013 PMC6619547

[b99] Bernard C. Lecöns de physiologie expérimentale appliquée á la médecine, faites au Collége de France. J.B. Bailliére et fils, 296–313 (Librairies de l'academie Imperiale de Medecine, 1855).

[b100] Chen Y., Lin Y. C., Kuo T. W. & Knight Z. A. Sensory detection of food rapidly modulates arcuate feeding circuits. Cell 160, 829–841 (2015). 25703096 10.1016/j.cell.2015.01.033 PMC4373539

[b101] Raffan E. . A deletion in the canine POMC gene is associated with weight and appetite in obesity-prone labrador retriever dogs. Cell Metab. 23, 893–900 (2016). 27157046 10.1016/j.cmet.2016.04.012 PMC4873617

[b102] Koch M. . Hypothalamic POMC neurons promote cannabinoid-induced feeding. Nature 519, 45–50 (2015). 25707796 10.1038/nature14260 PMC4496586

[b103] Gropp E. . Agouti-related peptide-expressing neurons are mandatory for feeding. Nat. Neurosci. 8, 1289–1291 (2005). 16158063 10.1038/nn1548

[b104] Wu Q., Boyle M. P. & Palmiter R. D. Loss of GABAergic signaling by AgRP neurons to the parabrachial nucleus leads to starvation. Cell 137, 1225–1234 (2009). 19563755 10.1016/j.cell.2009.04.022 PMC2729323

[b105] Ghamari-Langroudi M. . G-protein-independent coupling of MC4R to Kir7.1 in hypothalamic neurons. Nature 520, 94–98 (2015). 25600267 10.1038/nature14051 PMC4383680

